# Incidence of Post-Thyroidectomy Hypoparathyroidism and Associated Preoperative and Intraoperative Risk Factors

**DOI:** 10.7759/cureus.56585

**Published:** 2024-03-20

**Authors:** Hadi Afandi Al-Hakami, Renad M Alsolamy, Baraa I Awad, Roaa M Mandora, Dalia Hamdan, Rakan Alzahrani, Yousef Alaqsam, Mohammed Al-Garni

**Affiliations:** 1 College of Medicine, Department of Otolaryngology-Head & Neck Surgery, King Saud bin Abdulaziz University for Health Sciences, King Abdullah International Medical Research Center/Ministry of the National Guard - Health Affairs, Jeddah, SAU; 2 College of Medicine, King Saud bin Abdulaziz University for Health Sciences, King Abdullah International Medical Research Center, Jeddah, SAU; 3 Faculty of Medicine, Umm Al-Qura University, Makkah, SAU

**Keywords:** hypocalcemia, thyroid papillary cancer, thyroidectomy complications, hypoparathyroidism, thyroidectomy

## Abstract

Introduction: Thyroidectomy technique and extent are related to parathyroid injury and hypoparathyroidism. Total thyroidectomy is one of the most commonly performed endocrine surgeries, and the majority of patients recover completely without any complications. However, persistent hypoparathyroidism is the most prevalent long-term consequence following total thyroidectomy. While it is seldom deadly, it can cause severe morbidity for the patient and raise healthcare expenses.

Methods: This retrospective cohort study was conducted at King Abdulaziz Medical City, Jeddah, Saudi Arabia. We included all confirmed thyroid cancer cases that underwent thyroidectomy with or without neck dissection between July 2016 and August 2022. The data was collected from a chart review of the electronic medical record system (BEST-care), and a data collection sheet was utilized. SPSS version 26 was used to analyze the data.

Results: A total of 192 patients undergoing thyroid surgery were enrolled. One hundred forty-three (74.5%) were females and the mean age of participants was 45.29 ± 16.88 years. Most patients, 170 (88.5%), had a papillary histological type, and total thyroidectomy was performed in 150 (78.1%). A significant association was found between the type of surgery and postoperative hypoparathyroidism (p=<0.05*). In addition, hypocalcemia was seen in 147 (76.6%) of the patients. Postoperative hypoparathyroidism was significantly higher among patients who had asymptomatic postoperative hypocalcemia and those who received IV calcium gluconate (p=<0.05*). Moreover, postoperative hypocalcemia, hypomagnesemia, and hyperphosphatemia were significantly associated with postoperative hypoparathyroidism (p=<0.05*).

Conclusion: The incidence of postoperative hypoparathyroidism is significantly higher among patients who underwent total thyroidectomy and had a normal level of preoperative parathyroid hormone (PTH) and magnesium (Mg) levels. Identifying these factors is a crucial step to minimize the occurrence of such complications.

## Introduction

Total thyroidectomy is one of the most commonly performed endocrine surgeries, and the majority of patients recover completely without any complications. However, hypoparathyroidism is one of the most prevalent consequences following total thyroidectomy. While it is seldom deadly, it can cause severe morbidity for the patient and raise healthcare expenses [1].

Hypoparathyroidism is a rare endocrine disorder defined by low or inadequate amounts of circulating parathyroid hormone (PTH), which results in hypocalcemia (low serum calcium levels). Hypocalcemia can be acute, with paresthesia and neuromuscular instability, or chronic, with seizures, cataracts, ectopic calcifications, aberrant teeth, abnormal renal function, and mental diseases [1].

The surgical technique and the extent of thyroidectomy are related to parathyroid injury and hypoparathyroidism. The glands should be identified in situ, carefully manipulated and preserved, as well as their vascularization. In the case of incidental removal, routine autotransplantation is advocated. Low calcium levels, identification of fewer than two parathyroid glands at surgery, reoperation for bleeding, Graves’ disease, and heavier thyroid specimens were considered independent predictors of permanent hypocalcemia.

Intraoperative PTH measurements allow the early detection of hypocalcemia [2]. Worldwide and well-known, in 2020, the age-standardized incidence rates of thyroid cancer were 10.1 per 100000 women and 3.1 per 100000 men, and age-standardized mortality rates were 0.5 per 100000 women and 0.3 per 100000 men. While death rates varied little between different environments, bigender incidence rates were five times greater in high and very high Human Development Index countries than in low and medium Human Development Index countries [3]. Individuals who have had neck surgery are thought to experience transient post-surgical hypoparathyroidism in 25.4-83% of cases, while persistent post-surgical hypoparathyroidism is expected to occur in only 0.12-4.6% of patients [4].

PTH levels are the most common biochemical markers that are measured to predict postoperative hypocalcemia. However, there is inconsistent evidence linking PTH levels with postoperative hypocalcemia [5-7]. Furthermore, there is a debate on the most important cut-off for PTH postoperatively and the proper time for measuring PTH after surgery. Studies revealed that measuring PTH levels 1 hour after surgery might be predictive of postoperative hypocalcemia [6, 8-12]. Meanwhile, other studies suggested that more than 44% or 60% reduction in PTH levels is predictive of transient low calcium levels [13-16].

Despite the well-known effect of parathyroid autotransplantation in preventing postoperative hypoparathyroidism. Recent studies are questioning its value. In fact, the rate of PtHP following total thyroidectomy was not affected based on parathyroid autotransplantation in Lorente-Poch et al.’s study. Hypoparathyroidism was documented with a rate of 5.3% in patients with incidental parathyroidectomy without autotransplantation and 7.3% in patients with parathyroid autotransplantation [17]. The option of parathyroid autotransplantation could be appealing to patients especially in whom hypoparathyroidism is severe as the idea of treatment that can normalize calcium levels and restore gland function is promising to these patients.

According to Orloff et al., hypoparathyroidism has been linked to the extent of the surgery and performance of neck dissection; however, there is insufficient data regarding the association between patient comorbidities and post-thyroidectomy with/without neck dissection hypoparathyroidism. Also, there is limited reported data regarding the prevalence and incidence of hypoparathyroidism in Saudi Arabia [15].

Therefore, in this study, we aimed to first measure the incidence of postoperative hypoparathyroidism following thyroid cancer surgery at a tertiary hospital in Saudi Arabia. Second, to explore patient and surgery-related variables that may be significantly associated with the development of hypoparathyroidism.

## Materials and methods

Study design, setting, and period

This retrospective cohort study was conducted at the King Abdulaziz Medical City (KAMC), Jeddah, Saudi Arabia. A data collection sheet was used to collect information from the electronic medical records (BEST-Care System) about patients who underwent total thyroidectomy or completed thyroidectomy. The data were collected for cases from July 2016 to August 2022, with 192 electronic medical records involved in the study.

Study subjects and data collection

The inclusion criteria for the study included all patients with thyroid cancer that was confirmed by fine needle aspiration or histological examination who were diagnosed from July 2016 to the end of August 2022 at the KAMC, Jeddah, Saudi Arabia. Patients with missing postoperative follow-up data were excluded from the analysis.

The data was collected from the chart review of the electronic medical record system (BEST-care) and a data collection sheet was utilized. The data collection sheet was tailored to retrieve patients' demographic information, medical history, surgical details, histological data, and results of blood tests (such as preoperative/postoperative calcium level, PTH level, and magnesium level). The cut-off for PTH level to define hypoparathyroidism was less than 24 pg/mL.

Data management and statistical analysis

All data was collected by utilizing a data collection sheet. Data was entered into Microsoft Excel 2016 and then transferred to SPSS version 26 to be analyzed statistically. To assess the relationship between variables, qualitative data was presented as numbers and percentages, and the Chi-squared test (χ2) was used. Quantitative data was presented as mean and standard deviation (Mean ± SD), and non-parametric variables were tested using the Mann-Whitney and independent sample t-tests according to data normality. Correlation analysis was performed using Spearman's test and the odds ratio was calculated at a confidence interval (CI) of 95%. A p-value of less than 0.05 was regarded as statistically significant and a p-value of less than 0.05 was considered statistically significant.

Ethical considerations

The research was approved by the Institutional Review Board (IRB) from King Abdullah International Medical Research Center (KAIMRC), Jeddah, Saudi Arabia (IRB approval number: JED-22-427780-183248). Informed consent from patients was not required for this study because it was non-interventional and solely a retrospective examination of data from ordinary clinical practice. No names or any private information were collected. The data was saved in the workplace computer and protected by a password. Only the principal investigator and co-investigators had access to the data.

## Results

**Figure 1:**
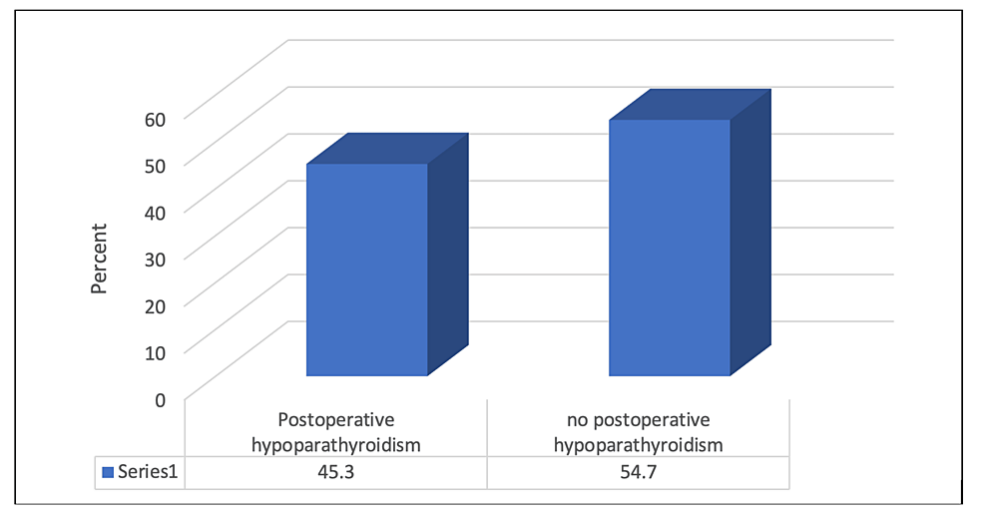
Percentage distribution of the prevalence of postoperative hypoparathyroidism

A total of 192 patients undergoing thyroid surgery for thyroid cancer between July 2016 and August 2022 were enrolled in this study. Most patients, 143 (74.5%), were females. The mean age of the studied patients was 45.29 ± 16.88 years. Postoperative hypoparathyroidism was documented in 45.3% of patients. As for the tumor characteristics, most patients, 170 (88.5%), had a papillary histological type, 36 (18.8%) had an extrathyroidal extension, 105 (54.7%) had a unifocal tumor and the majority 156 (81.3%) had a tumor of stage 1. Almost one-third of patients 65 (33.9%) had lymph node metastasis, 42 (21.9%) had an extra-capsular extension, and 49 (25.5%) had > one parathyroid gland removed as an unintentional parathyroidectomy. Hashimoto thyroiditis or lymphocytic thyroiditis was seen in 66 (34.4%). As for the management, total thyroidectomy was performed in 150 (78.1%) patients, and neck dissection was done in 70 (36.5%) of the studied patients. A significant association was found between the type of surgery and the occurrence of postoperative hypoparathyroidism as patients who underwent total thyroidectomy without neck dissection were most likely to have postoperative low PTH levels (p=<0.05). Further details are in Figure 1 and Table 1.

**Table 1:** Relationship between the prevalence of postoperative hypoparathyroidism and patients' demographics tumor characters, surgical procedure, neck dissection and if parathyroid gland identified during surgery (N = 192)

Variable	Total	Postoperative hypoparathyroidism		χ2	p-value
	No. (%)	Yes (No.%)	No (No. %)		
Age (years)	45.29 ± 16.88	44.32 ± 16.65	46.09 ± 17.1*	0.72	0.742
Tumor size (mm)	2.92 ± 2.29	3.04 ± 2.45	2.81 ± 2.14**	0.41	0.681
Size of largest metastasized lymph node (mm)	2.4 ± 1.98	2.66 ± 2.28	2.01 ± 1.36**	0.34	0.73
Total taken lymph node	17.75 ± 20.64	20.12 22.05	15 ± 18.75**	1.47	0.141
Number of metastasized lymph node	4.63 ± 5.98	5.33 ± 6.3	3.88 ± 5.59**	0.84	0.401
Gender				2.99	0.084
Male	49 (25.2)	17 (19.5)	32 (30.5)		
Female	143 (74.5)	70 (80.5)	73 (69.5)		
Histological type				2.32	0.313
Papillary	170 (88.5)	80 (92)	90 (85.7)		
Follicular	12 (6.3)	3 (3.4)	9 (8.6)		
Others	10 (5.2)	4 (4.6)	6 (5.7)		
Extrathyroidal extension				3.76	0.152
No	4 (2.1)	0 (0.0)	4 (3.8)		
Yes	36 (18.8)	15 (17.2)	21 (20)		
No or not mentioned	152 (79.2)	72 (82.8)	80 (76.2)		
Tumor focality				0.02	0.866
Unifocal	105 (54.7)	47 (54)	58 (55.2)		
Multifocal	87 (45.3)	40 (46)	47 (44.8)		
Stage				0.07	0.961
1	156 (81.3)	70 (80.5)	86 (51.9)		
2	32 (16.7)	15 (17.2)	17 (16.2)		
3	4 (2.1)	2 (2.3)	2 (1.9)		
Lymph node metastasis				1.94	0.164
Yes	65 (33.9)	34 (39.1)	31 (29.5)		
No	127 (66.1)	53 (60.9)	74 (70.5)		
Extra-capsular extension				2.88	0.237
Not mentioned	34 (17.7)	11 (12.6)	23 (21.9)		
Yes	42 (21.9)	21 (24.1)	21 (20)		
No	116 (60.4)	55 (63.2)	61 (58.1)		
Un-intentional para thyroidectomy				5.12	0.077
Portion of parathyroid	19 (9.9)	8 (9.2)	11 (10.5)		
No	124 (64.6)	50 (57.3)	74 (70.5)		
> one parathyroid	49 (25.5)	29 (33.3)	20 (19)		
Hashimoto thyroiditis or lymphocytic thyroiditis				1.97	0.373
Not mentioned	2 (1)	0 (0.0)	2 (1.9)		
Yes	66 (34.4)	32 (36.8)	34 (32.4)		
No	124 (64.6)	55 (63.2)	69 (65.7)		
Surgical procedure				10.03	0.002
Total thyroidectomy	150 (78.1)	77 (88.5)	73 (69.5)		
Completion thyroidectomy	42 (21.9)	10 (11.5)	32 (30.5)		
Central neck dissection				4.81	0.028
Yes	70 (36.5)	39 (44.8)	31 (29.5)		
No	122 (63.5)	48 (55.2)	74 (70.5)		
Parathyroid gland identified during surgery				2.41	0.21
Yes	126 (65.6)	52 (59.8)	74 (70.5)		
No	66 (34.4)	35 (40.2)	31 (29.5)		

Participants’ background comorbidities included obesity in 35 (18.2%) and kidney disease in five (2.6%). Either parathyroid adenoma or concurrent malignancy was document in three (1.6%) of patients. In addition, postoperative hoarseness was seen in 16 (8.4%) of patients and vocal cord paralysis seen via fiberoptic laryngoscopy (FOL) was evident in four (2.1%). As for postoperative laboratory findings, hypocalcemia was seen in 147 (76.6%) of the patients. The most common symptom of post-op hypocalcemia was numbness in 19 (10.4%), while 162 (88.5%) of patients were asymptomatic. About 14 (14.3%) of patients had IV calcium gluconate administration. It was found that postoperative hypoparathyroidism was significantly higher among patients who had no symptoms of post-op hypocalcemia and among those who had IV calcium gluconate given (p=<0.05). On the other hand, no significant relationship was found between the prevalence of postoperative hypoparathyroidism and the presence of parathyroid adenoma, Grave’s disease, concurrent malignancy, obesity, kidney disease, postoperative hoarseness, or FOL (p=>0.05), Table 2 shows further details.

**Table 2:** Relationship between the prevalence of postoperative hypoparathyroidism and parathyroid adenoma, Grave’s disease, obesity, concurrent malignancy, obesity, kidney disease postoperative hoarseness, FOL, post-op symptoms of hypocalcemia, and having IV calcium gluconate (No = 192)

Variable	Total	Postoperative hypoparathyroidism		χ2	p-value
	No. (%)	Yes (No.%)	No (No. %)		
Parathyroid adenoma	3 (1.6)	2 (2.3)	1 (1)	0.56	0.454
Grave’s disease	1 (0.5)	0 (0.0)	1 (1)	0.83	0.361
Concurrent malignancy	3 (1.6)	2 (2.3)	1 (1)	0.56	0.454
Obesity	35 (18.2)	19 (21.8)	16 (15.2)	1.39	0.238
Kidney disease	5 (2.6)	4 (4.6)	1 (1)	2.49	0.114
Postoperative hoarseness	16 (8.4)	9 (10.5)	7 (6.7)	0.88	0.346
Fiberoptic laryngoscopy showed vocal cord paralysis (FOL)	4 (2.1)	0 (0.0)	4 (100)	3.38	0.184
Postoperative symptoms of hypocalcemia				14.13	0.003
Tingling	1 (0.5)	1 (1.3)	0 (0.0)		
Numbness	19 (10.4)	15 (19)	4 (3.8)		
Muscle spasm/cramps	1 (0.5)	1 (1.3)	0 (0.0)		
No symptoms	162 (88.5)	62 (78.5)	100 (96.2)		
IV calcium gluconate given	27 (14.3)	23 (26.7)	4 (3.9)	20.99	<0.001

The mean values of pre- and postoperative laboratory data. It was observed that patients who had postoperative hypoparathyroidism had a significantly lower mean value of post-op adjusted calcium, and a significantly higher mean value of post-op Mg level or post-op phosphorus level (p=<0.05). Further details are in Table 3. This finding could be attributed to the fact that patient who had postoperative hypoparathyroidism was treated with calcium.

**Table 3:** Relationship between the prevalence of postoperative hypoparathyroidism and blood loss and pre- and postoperative laboratory data (No = 192)

Variable	Total	Postoperative hypoparathyroidism		Mann-Whitney test	p-value
	Mean ± SD	Yes Mean ± SD	No Mean ± SD		
Blood loss (mL)	108.75 ± 226.42	109.64 ± 190.53	108.02 ± 252.96	1.81	0.069
Preoperative					
Pre-op adjusted calcium level (mmol/L)	2.28 ± 0.11	2.29 ± 0.11	2.27 ± 0.11	0.86	0.385
Pre-op PTH level (pg/mL)	77.74 1± 05.68	65.91 ± 55.43	87.43 ± 132.98	1.47	0.14
Pre-op Mg level (mmol/L)	1.24 ± 5.32	0.96 1±.08	1.48 ± 7.14	0.86	0.387
Pre-op phosphorus level (mmol/L)	1.13 ± 0.18	1.14 ± 0.18	1.12 ± 0.18	0.79*	0.429
Postoperative					
Post-op adjusted calcium (mmol/L)	2.05 ± 0.21	1.99 ± 0.2	2.11 ± 0.2	5.47	<0.001
Post-op Mg level (mmol/L)	1.19 ± 6.24	1.69 ± 9.25	0.78 ± 0.22	3.78	<0.001
Post-op phosphorus level (mmol/L)	1.59 ± 0.38	1.81 ± 0.4	1.4 ± 0.23	7.39	<0.001

Low levels of preoperative calcium, PTH, magnesium, and phosphorus were seen in 36 (18.8%), 32 (16.7%), 27 (14.1%), and three (1.6%) of patients, respectively. Furthermore, postoperative hypocalcemia and hypomagnesemia were documented in 147 (76.6%) and 84 (43.8%) of patients, respectively. While none had a postoperative low phosphorus level. Surprisingly, the prevalence of postoperative hypoparathyroidism was significantly higher among patients who had a normal level of preoperative PTH and Mg levels. While postoperative hypocalcemia, hypomagnesemia, and hyperphosphatemia were significantly associated with postoperative hypoparathyroidism (p=<0.05). Further details are in Table 4. Furthermore, a significant positive correlation was found between post-op PTH level and postoperative Ca, Mg, and phosphorus (p=<0.05) (Table 5).

**Table 4:** Relationship between the prevalence of postoperative hypoparathyroidism and levels of pre- and postoperative laboratory data according to reference values (No = 192). Lab values were taken at day 1 post-op.

Variable	Total	Postoperative hypoparathyroidism		χ2	p-value
	No. (%)	Yes No. (%)	No Mean ± SD		
Preoperative					
Pre-op adjusted calcium level (Reference value: 2.2 to 2.54 mmol/L)				1.22	0.542
Low	36 (18.8)	14 (16.1)	22 (21)		
Normal	153 (79.7)	71 (81.6)	82 (78.1)		
High	3 (1.6)	2 (2.3)	1 (1)		
Pre-op PTH level (Reference value: 24-114 pg/mL)				13.65	0.001
Low	32 (16.7)	24 (27.6)	8 (7.6)		
Normal	132 (68.8)	52 (59.8)	80 (76.2)		
High	28 (14.6)	11 (12.6)	17 (16.2)		
Pre-op Mg level (Reference value: 0.72-0.96 mmol/L)				6.09	0.048
Low	27 (14.1)	11 (12.6)	16 (15.2)		
Normal	157 (81.8)	69 (79.3)	88 (83.8)		
High	8 (4.2)	7 (8)	1 (1)		
Pre-op phosphorus level (Reference value: 0.75 to 1.51 mmol/L)				0.21	0.9
Low	3 (1.6)	1 (1.1)	2 (1.9)		
Normal	185 (96.4)	84 (96.6)	101 (96.2)		
High	4 (2.1)	2 (2.3)	2 (1.9)		
Postoperative					
Post-op adjusted calcium (Reference value: 2.2 to 2.54 mmol/L)				6.77	0.034
Low	147 (76.6)	74 (85.1)	73 (69.5)		
Normal	43 (22.4)	12 (13.8)	31 (29.5)		
High	2 (1)	1 (1.1)	1 (1)		
Post-op Mg level (Reference value: 0.72-0.96 mmol/L)				9.19	0.01
Low	84 (43.8)	48 (55.2)	36 (34.3)		
Normal	99 (51.6)	37 (42.5)	62 (59)		
High	9 (4.7)	2 (2.3)	7 (6.7)		
Post-op phosphorus level (Reference value: 0.75 to 1.51 mmol/L)				41.25	<0.001
Normal	93 (48.4)	20 (23)	73 (69.5)		
High	99 (51.6)	67 (77)	32 (30.5)		

**Table 5:** Spearman's correlation analysis between post-op PTH level and postoperative Ca, Mg, and phosphorus PTH: parathyroid hormone

Variable	Post-op PTH level	
	r	p-value
Post-op adjusted calcium	0.44	<0.001
Post-op Mg level	0.26	<0.001
Post-op phosphorus level	0.57	<0.001

Multivariate logistic regression analysis was done to assess the risk factors (independent predictors) of postoperative hypoparathyroidism among studied patients. It was found that having no neck dissection, total thyroidectomy, or a high mean post-op phosphorus level were risk factors (independent predictors) of postoperative hypoparathyroidism (Table 6).

**Table 6:** Multivariate logistic regression analysis of risk factors of postoperative hypoparathyroidism among studied patients

Variable	Wald	B	p-value	Odds Ratio (CI:95%)
Neck dissection	0.93	5.68	0.017	2.55 (1.18-5.53)
Surgical procedure	1.04	4.6	0.032	2.83 (1.09-7.33)
IV calcium gluconate given	0.13	0.16	0.682	0.87 (0.46-1.56)
Post-op adjusted calcium	0.72	0.65	0.42	2.07 (0.35-1.17)
Post-op Mg level	0.02	0.01	0.892	0.97 (0.7-1.35)
Post-op phosphorus level	4.63	30.63	<0.001	1.23 (1.7-2.34)

## Discussion

It is estimated that 25.4-83% of individuals who have had neck surgery may develop transient post-surgical hypoparathyroidism, but only 0.12-4.6% of patients are predicted to have permanent post-surgical hypoparathyroidism [4]. This research aimed to evaluate the risk factors associated with low PTH levels following thyroid cancer surgery. Our goal was to identify patient- and surgery-related variables that might be strongly linked to the development of hypoparathyroidism. Firstly, parathyroid failure after thyroidectomy is defined as PTH ≤10 pg/mL with hypocalcemia symptoms [18]. According to the available literature, the incidence of hypoparathyroidism post-thyroidectomy happens more often in the female gender which also shows similar results in our study [1,12,19].

Along with Bassam et al. and Zambudio et al., we have reached similar outcomes regarding age, which does not affect the parathyroid gland and its function after the surgery [20-21]. Furthermore, we found that the incidence of transient and permanent hypoparathyroidism is directly proportional to the extent of the surgical procedure, which also was reported in previous literature [1,19,22].

Also, an independent connection between the occurrence of postoperative hypoparathyroidism and combined lymph node dissection along with the tumor diameter of the thyroid gland, a second operation, and preoperative hypocalcemia in a three-year retrospective investigation was observed in Ru et al.’s study [23]. This was also observed in our study along with other literature [1].

Neck dissection of the central lymph nodes often done with malignant thyroid disease may significantly elevate the likelihood of direct damage to the parathyroid glands or disrupt blood circulation [24]. Consequently, many studies recommend the use of nano-carbon or other color reagents as a protective measure to visually distinguish the parathyroid glands during neck dissection in an attempt to minimize the disruption [25]. In our study, we found 44.8% of patients had hypoparathyroidism post-neck dissection with a p-value of 0.028.

A 2018 meta-analysis found that certain technical elements during surgery may increase the likelihood of having hypocalcemia [15]. Additionally, based on the available literature, hypoparathyroidism may occur despite the preservation of parathyroids, and some studies indicate that doing a systematic search during surgery increases the chance of transient hypocalcemia [26]. This also corresponds with our findings.

However, during our investigation, we were unable to find any association between preoperative medical illnesses such as parathyroid adenoma, Grave’s disease, kidney disease, obesity, and concurrent malignancies with postoperative hypoparathyroidism due to insufficient data. Nevertheless, Edafe et al. in his systematic review and meta-analysis concluded that patients with Grave’s disease are more likely to develop postoperative hypothyroidism when compared to others [12]. This could be explained by Karamanakos et al.’s study which found that some cases of Graves' disease and recurrent operations might be connected to the existence of adhesions between the capsule of the thyroid gland and the parathyroid gland [27].

On the other hand, postoperative hypocalcemia has been identified as the most common complication following thyroidectomy in routine clinical practice, and in its severe forms, it can cause prolonged hospitalization and impact the quality of life [12]. Hypocalcemia is characterized by a decrease in the concentration of calcium in the bloodstream. It can also manifest through symptoms such as numbness, tingling sensations, and muscular cramps. Even though during our infestation, we found 78.5% of our population had hypoparathyroidism with no symptoms of hypocalcemia. Furthermore, the literature shows that preoperative low or low-normal levels of PTH are significantly associated with hypocalcemia post-surgery [12,28]. Thus, Edafe et al. proposed that the measured level of calcium, 25-hydroxyvitamin D, and PTH pre-operatively may serve as predictors for their respective levels post-surgery [29]. However, it is not recommended to routinely screen PTH and 25-hydroxyvitamin D pre-operatively when calcium level is within the normal range.

While the parathyroid gland requires a consistent and adequate blood supply to function properly, we could not ascertain a correlation between intraoperative blood loss and postoperative parathyroid dysfunction [30].

This study attempted to enhance the understanding of the variables of post-thyroidectomy hypoparathyroidism and emphasize the prediction of the progression of this problem. There are a few challenges and obstacles to this study. First, it was conducted in one center with a small sample size. Second, it was retrospective. Finally, the discrepancies in the criteria used to define hypoparathyroidism are possibly impacted by institutional bias. To address these constraints, we seek to conduct a forthcoming prospective multicenter investigation. In addition, in our study, we were unable to identify any association between patients’ clinical background and the development of postoperative hypothyroidism due to insufficient data. This highlights the need for larger studies to pinpoint the value of these factors in predicting postoperative hypothyroidism.

## Conclusions

The incidence of postoperative hypoparathyroidism is significantly higher among patients who underwent total thyroidectomy and had a normal level of preoperative PTH and Mg levels. Identifying these factors is a crucial step to minimize the occurrence of such complications.
